# Draft Genome Sequence of Clostridium estertheticum CEST001, Belonging to a Novel Subspecies of *C. estertheticum*, Isolated from Chilled Vacuum-Packed Lamb Meat Imported to Switzerland

**DOI:** 10.1128/MRA.00806-20

**Published:** 2020-08-13

**Authors:** Joseph Wambui, Nicole Cernela, Marc J. A. Stevens, Roger Stephan

**Affiliations:** a Institute for Food Safety and Hygiene, Vetsuisse Faculty, University of Zurich, Zurich, Switzerland; University of Rochester School of Medicine and Dentistry

## Abstract

We present the draft genome sequence of Clostridium estertheticum strain CEST001. The genome is 4.8 Mbp long with a GC content of 30.6%. The digital DNA-DNA hybridization values against four *C. estertheticum* strains indicate that *C. estertheticum* CEST001 belongs to a novel subspecies of *C. estertheticum*.

## ANNOUNCEMENT

Clostridium estertheticum is a spore-forming anaerobic psychrophilic bacterium causing blown-pack spoilage (BPS) in chilled vacuum-packed meat (1). Here, we determined the draft genome sequence of β-hemolytic *C. estertheticum* CEST001, isolated from chilled vacuum-packed lamb meat imported from New Zealand to Switzerland (2).

Isolation of the strain was carried out anaerobically at 4°C in a multistep process involving ethanol and lysozyme treatment followed by enrichment in peptone yeast glucose starch medium for 3 weeks and plating and incubation for 3 weeks on Columbia agar supplemented with 5% defibrinated sheep blood (CBA). The strain was further subcultured on CBA for 2 weeks at 4°C for genomic DNA extraction.

Genomic DNA was isolated using the DNA blood and tissue kit (Qiagen, Hombrechtikon, Switzerland). Sequencing libraries were prepared using Nextera DNA Flex chemistry (Illumina, San Diego, CA, USA), and the resulting transposome-based libraries were sequenced on a MiniSeq sequencer (Illumina). The sequencing output was 277 Mbp paired-end reads of 150 to 300 bp. Reads were checked for quality using the software package FastQC 0.11.7 (3) and then assembled using the SPAdes 3.0-based software (4) Shovill 1.0.4 (https://github.com/tseemann/shovill). The assembly was filtered, retaining contigs of >500 bp. The genome was annotated using the NCBI Prokaryotic Genome Annotation Pipeline (5) and the RAST pipeline (6). Default parameters were used for all software and Web servers.

The genome was assembled into 65 contigs and comprises 4.8 Mbp with a GC content of 30.6%, 4,678 genes, and 4,563 coding sequences. The genome coverage, contig *N*_50_ value, and contig *L*_50_ value are 50.0×, 222,559 bp, and 8, respectively. The 16S rRNA gene sequence was determined *in silico* (7) and used in the strain identification using the 16S-based identification tool (7). The strain was identified as *C. estertheticum*. The results were validated by whole-genome sequencing (WGS) using digital DNA-DNA hybridization (dDDH) (8) and average nucleotide identity (ANI) (9) against the WGS of four *C. estertheticum* strains (10–12). ANI and dDDH values for the identification of species ranged from 96.6 to 96.8% and 81.3 to 82.9%, respectively, which are above the 95% and 70% thresholds, respectively (8, 13, 14). However, the dDDH value for the identification of subspecies ranged from 32.1 to 34.1%, which is below the 79% threshold (8). This indicates that *C. estertheticum* CEST001 belongs to a novel subspecies of *C. estertheticum*.

tRNAscan-SE (15) identified 89 tRNAs. RAST (6) identified 104 RNAs and 34 features for antibiotic and metal tolerance. VFDB 2019 (16) predicted 32 potential virulence factors, but the strain was predicted to be a nonhuman pathogen by PathogenFinder (17).

The present genome sequence adds to the short list of publicly available *C. estertheticum* genome sequences.

### Data availability.

This whole-genome shotgun project has been deposited at DDBJ/ENA/GenBank under the accession no. JABEYB000000000. The version described in this paper is version JABEYB010000000. The raw sequencing reads have been deposited in the SRA under the accession no. SRR11775223.
